# Auditory Nerve Fiber Discrimination and Representation of Naturally-Spoken Vowels in Noise

**DOI:** 10.1523/ENEURO.0474-21.2021

**Published:** 2022-02-11

**Authors:** Amarins N. Heeringa, Christine Köppl

**Affiliations:** Cluster of Excellence “Hearing4all” and Research Centre Neurosensory Science, Department of Neuroscience, School of Medicine and Health Science, Carl von Ossietzky University Oldenburg, Oldenburg 26129, Germany

**Keywords:** consonant-vowel-consonant logatomes, ICRA1 noise, Mongolian gerbil, OLLO speech material database, temporal coding

## Abstract

To understand how vowels are encoded by auditory nerve (AN) fibers, a number of representation schemes have been suggested that extract the vowel’s formant frequencies from AN-fiber spiking patterns. The current study aims to apply and compare these schemes for AN-fiber responses to naturally-spoken vowels in a speech-shaped background noise. Responses to three vowels were evaluated; based on behavioral experiments in the same species, two of these were perceptually difficult to discriminate from each other (/e/ vs /i/), and one was perceptually easy to discriminate from the other two (/a:/). Single-unit AN fibers were recorded from ketamine/xylazine-anesthetized Mongolian gerbils of either sex (*n* = 8). First, single-unit discrimination between the three vowels was studied. Compared with the perceptually easy discriminations, the average spike timing-based discrimination values were significantly lower for the perceptually difficult vowel discrimination. This was not true for an average rate-based discrimination metric, the rate d-prime (d’). Consistently, spike timing-based representation schemes, plotting the temporal responses of all recorded units as a function of their best frequency (BF), i.e., dominant component schemes, average localized interval rate, and fluctuation profiles, revealed representation of the vowel’s formant frequencies, whereas no such representation was apparent in the rate-based excitation pattern. Making use of perceptual discrimination data, this study reveals that discrimination difficulties of naturally-spoken vowels in speech-shaped noise originate peripherally and can be studied in the spike timing patterns of single AN fibers.

## Significance Statement

Understanding speech in noisy environments is an everyday challenge. This study investigates how single auditory nerve (AN) fibers, recorded in the Mongolian gerbil, discriminate and represent naturally-spoken vowels in a noisy background approximating real-life situations. Neural discrimination metrics were compared with the known behavioral performance by the same species, comparing easy to difficult vowel discriminations. A spike-timing-based discrimination metric agreed well with perceptual performance, while mean discharge rate was a poor predictor. Furthermore, only spike-timing-based, but not the rate-based, representation schemes revealed peaks at the formant frequencies, which are paramount for perceptual vowel identification and discrimination. This study reveals that vowel discrimination difficulties in noise originate peripherally and can be studied in the spike-timing patterns of single AN fibers.

## Introduction

Vocal communication is indispensable for the survival and well-being of many species, including mammals. Speech is one of the more complex of such vocalizations that needs to be processed by the central auditory system. Nonetheless, humans, as well as behaviorally trained animals, are still well able to extract critical features of speech signals. Individuals can discriminate between different syllables or logatomes, short nonsensical pseudowords of one or two syllables, even when presented in challenging background sounds (Sinnott et al., 1997; Jürgens and Brand, 2009). This suggests that the mammalian auditory system is proficient at processing such temporally and spectrally complex signals.

Speech can be decomposed into vowels and consonants, in which vowels are spectrally, and consonants are temporally, more complex. In this study, we focused on the vowels. A vowel’s spectrum comprises harmonics of the fundamental frequency (f0), which is the voice pitch, and two to five formant frequencies (f1–f5), which are global regions of maximum energy in the vowel spectrum (Diehl, 2008). The formant frequencies differ between different vowels, whereas the f0 differs between speakers, but is similar between vowels spoken by the same speaker. For perceptual vowel discrimination, the formant frequencies are paramount; for example, whispered vowels, lacking f0, remain intelligible and discriminable. Hence for vowels to be discriminated, information on their formant frequencies needs to be encoded in the spike patterns of the auditory nerve (AN), the gateway between sensory hair cells of the inner-ear cochlea and the central auditory system.

To understand how the formant frequencies are encoded by the AN, previous research has suggested a number of analysis methods, or representation schemes, that can extract formant frequencies from AN-spiking patterns. Vowel representation schemes for the AN include rate-based excitation patterns (Sachs and Young, 1979), dominant component schemes (Delgutte and Kiang, 1984a), averaged localized synchronized rate (ALSR) plots (Young and Sachs, 1979), and fluctuation profiles (Carney, 2018). Applying such representation schemes to the AN response to vowels presented in background noise, as well as to vowels presented at moderately high sound levels, results in degradation of neural formant representation, more in some schemes than in others (Young and Sachs, 1979; Sachs et al., 1983; Delgutte and Kiang, 1984b). Furthermore, in the search for such representation schemes, synthetic vowels were often used, in which formant frequencies can coincide exactly with an f0 harmonic and spectral changes over time do not occur. In synthesized vowels, a particular feature can be changed independently of others, to study its importance in vowel representation. For example, by applying a range of f2 frequencies, Conley and Keilson (1995) measured the minimal frequency difference that is represented in a rate-based excitation pattern. In addition, it has been shown that synthetic vowels presented in a consonant-vowel construct show improved rate representation in the transition between consonant to vowel (Sinex and Geisler, 1983). Naturally-spoken vowels, where the spectrum is rarely steady state over >0.1 s, however, pose an additional challenge to the representation schemes, especially when presented in background noise.

Ultimately, vowel representation in the AN only needs to be as good as the ability to perceptually discriminate between two highly similar vowels. Indeed, some vowels are perceptually easier to discriminate from each other than others, which is related to the degree of similarity in the formant frequencies (Jürgens and Brand, 2009). Therefore, knowledge of the discrimination performance between vowels provides crucial guidance about important vowel features and representation schemes, especially in challenging listening conditions.

Here, we probed the neural representation of naturally-spoken vowels from the Oldenburg Logatome (OLLO) database of speech material (Meyer et al., 2010) presented in a speech-shaped background noise, by applying previously developed representation schemes. Vowels were selected based on psychophysical behavioral experiments using the same species (gerbil) and the same speech material. In these experiments, animals were trained to perform an oddball target detection paradigm on vowels. A perceptual map was generated from the animal’s behavioral response latencies using multidimensional scaling and shows difficult and easy discriminations as short and long distances, respectively. For the current study, we selected three vowels from this perceptual map. Thus, vowel discrimination could be compared between vowels that were easy and those that were difficult to perceptually distinguish by gerbils (Jüchter et al., 2021). Making use of perceptual discrimination data in challenging conditions, this study sheds light on the accuracy of different AN fiber representation schemes for revealing the encoding of naturally-spoken vowels in a speech-shaped background noise.

## Materials and Methods

### Animals

Single-unit AN recordings from eight young-adult (three to six months) Mongolian gerbils (*Meriones unguiculatus*) were used in this study. Animals weighed between 63 and 94 g, were of either sex, and were free from middle-ear infections. Animals were born at the University of Oldenburg animal facility and were group housed (two to four litter mates of the same sex per cage) in a controlled, quiet environment. Experiments were conducted in a custom-build sound-attenuating chamber. Animals were anesthetized with an intraperitoneal injection containing a mixture of ketamine (135 mg/kg; Ketamidor, WDT) and xylazine (6 mg/kg; Xylazin, Serumwerk) diluted in saline (0.9% NaCl). One-third of the initial ketamine/xylazine dose was provided hourly or on a positive hind-paw reflex. Rectal body temperature was maintained at 38°C by a homeothermic blanket (Harvard Apparatus). Gerbils received oxygen flowing onto the snout (1.5 l/min). The head of the animal was fixed in a bite bar (Kopf Instruments) and the right pinna was removed. The ear bar, containing a small speaker (IE 800, Sennheiser) and microphone (ER7-C, Etymotic Research), was sealed to the bony ear canal using petroleum jelly. A small opening in the bulla prevented the build-up of negative pressure in the middle ear cavity. To determine hearing thresholds before and during single-unit recordings, auditory brainstem responses (ABRs) to chirps (0.3–19 kHz, 4.2-ms duration, 300–500 repetitions) at a range of levels were recorded and visually evaluated. Experiments were terminated on an ABR threshold elevation of >20 dB, or on heart failure. All experimental procedures were performed in accordance with the animal ethics authorities of Lower Saxony, Germany (permit number AZ 33.19-42502-04-15/1990).

### Single-unit AN-fiber recordings

The brainstem was exposed through a craniotomy of the right occipital bone, followed by a duratomy and partial aspiration of the cerebellum. The AN was visualized by placing small balls of paper tissue (<0.5 mm in diameter), drenched in saline, between the brainstem and the temporal bone. Glass micropipette electrodes (GB120F-10, Science Products, pulled with a P-2000, Sutter Instruments) were filled with a 3 M KCl solution (21 to 44-MΩ impedance). Electrodes were advanced slowly through the AN bundle (6000 ULN inchworm motor controller and 6005 ULN handset, Burleigh), while playing a broadband-noise search stimulus (50–70 dB SPL) through the speaker, until a single unit was isolated. Neural signals were amplified (WPI 767, World Precision Instruments), filtered for line-frequency noise (50/60 Hz; Hum Bug, Quest Scientific), made audible through a speaker (MS2, TDT), visualized on an oscilloscope (SDS 1102CNL, SIGLENT Technologies), and digitized (RX6, TDT; 48 828 Hz sampling rate) before storage on a personal computer using custom-written MATLAB software.

Tone bursts near 10 dB above the most sensitive threshold (50 ms ON–180 ms OFF time, 5-ms cosine rise/fall times, five repetitions, 10–20 linear frequency steps spanning around 1.5 octave) were presented to determine the unit’s best frequency (BF). Subsequently, tone bursts at BF were presented at a range of levels (3 dB step size, 10 repetitions) to determine the unit’s threshold. When the unit was still stable after recording responses to the consonant-vowel-consonant (CVC) stimuli, 24 s of neural activity in the absence of acoustic stimuli were recorded to more accurately determine the unit’s spontaneous rate (SR). When this recording was not available (40.5% of units), SR was estimated through the silent trials of the rate-level function recordings. All stimuli were calibrated using the miniature microphone (ER7-C, Etymotic Research) sealed near the speaker in the ear bar, a microphone amplifier (MA3, TDT), and custom-made MATLAB software.

### CVC stimuli

**Figure 1.**
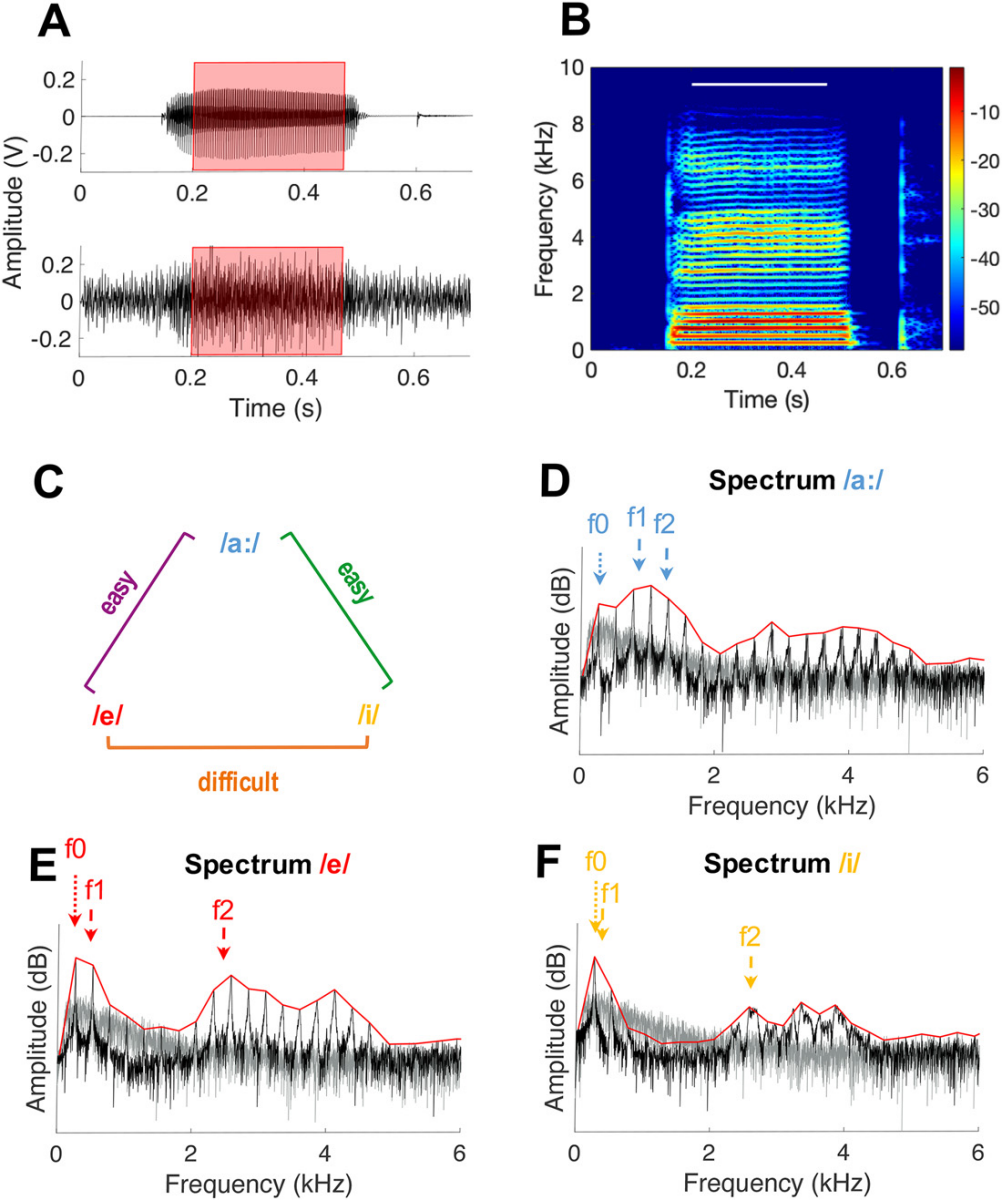
Characteristics of the three vowels used in the current study. ***A***, Pressure waveform of /bahb/ in quiet (upper panel) and in 5 dB SNR speech-shaped noise (lower panel). The vowel analysis window is highlighted in red. ***B***, Spectrogram of /bahb/ with the vowel analysis window indicated with the white bar. ***C***, The three vowels for which AN-fiber responses were recorded and their corresponding perceptual discriminability as shown by behavioral studies (Jüchter et al., 2021). Colors of the vowels and of each vowel comparison are similar to the colors used in the subsequent figures. ***D–F***, Spectral representation of the naturally-spoken vowels /a:/, /e/, and /i/, respectively. Vowels were derived from the OLLO speech material database and spoken by a female speaker, with /b/ as a flanking consonant. Spectra were generated from the stimulus in the vowel’s corresponding analysis window (see panels ***A***, ***B*** and Table 1). Fundamental (f0) and formant frequencies (f1 and f2) are indicated for each vowel. The spectral envelope of each vowel is indicated by a red line. Note that frequency of the vowel spectra is plotted on a linear scale to improve visualization of the f0 harmonics. The spectrum of the frozen ICRA1 speech-shaped noise, presented at 5 dB SNR, is plotted separately indicated in gray for all vowel spectra.

Vowels were presented in a CVC construct and derived from the OLLO speech-material database (Meyer et al., 2010). Figure 1*A* shows an example of the first 0.7 s of the stimulus waveform /bahb/ in quiet (Fig. 1*A*, upper panel) and in 5 dB signal-to-noise ratio (SNR) noise (Fig. 1*A*, lower panel). The spectrogram of the same stimulus, in quiet, is shown in Figure 1*B*. For each CVC, an analysis window was defined, which included the steady-state section of the vowel (Fig. 1*A*, red square, *B*, white bar). CVCs were selected based on a behavioral study in gerbils from the same colony that had demonstrated common and uncommon confusions between all vowels available in the database (Jüchter et al., 2021). Three vowels were selected from that study: two that were difficult to discriminate from each other (/e/ vs /i/) and one that was easy to discriminate from the other two (/a:/; Fig. 1*C*). The outer consonant was fixed at /b/, resulting in the following spoken logatomes: /behb/, /bieb/, and /bahb/. CVCs were spoken by a single female speaker who was a native German speaker (noted S01F in the OLLO database). The f0, as well as the length of the vowel, which was used as an analysis window, differed slightly between the three vowels (see Table 1). In a control experiment, the same logatomes were used but spoken by a native German male speaker (S06M in the OLLO database). All CVCs were calibrated according to the recorded transfer function of the acoustic set up using custom-made MATLAB software. Formant frequencies (f1 and f2) were derived from the vowel spectrum of the stimulus recorded with the microphone in the ear bar, i.e., the vowel spectrum of the stimulus delivered to the gerbil’s ear (Table 1; Fig. 1*D–F*).

**Table 1:** Fundamental and formant frequencies and length of the analysis windows of the presented vowels

	f0 (Hz)	f1 (Hz)	f2 (Hz)	Analysis window (ms)
F /e/	256	400	2450	268
F /i/	260	300	2575	233
F /a:/	257	850	1275	269
M /e/	107	300	1990	121
M /i/	109	220	2040	110
M /a:/	104	650	1150	143

Stimuli spoken by the female speaker are marked with F, stimuli spoken by the male speaker are marked with M.

The total recording time of one presentation of a logatome was fixed at 1.15 s. The logatome started after a delay of 125 ms, had an average length of 570 ms, and was presented at a fixed level of 65 dB SPL. Logatomes were presented against a speech-like background noise (ICRA1) derived from the ICRA database (Dreschler et al., 2001; Fig. 1*D–F*, gray traces). Calibrated noise was presented at a 5-dB SNR and mixed with the logatome before being presented through the same in-ear speaker. Each logatome was sequentially presented 60 times. Noise was frozen between repetitions, as well as between different units. A total of two different frozen noises were presented for one complete set of stimuli. Noise onset and offset were ramped with a 10-ms cosine squared window at the start and end of the recording time.

### Data analysis

Neural signals were bandpass filtered between 0.3 and 3 kHz. The threshold for spike detection was revisited offline for all recordings on a trial-by-trial basis, as spike amplitude could vary during recordings. Single-unit isolation was confirmed by checking interspike intervals, of which a large majority should be >1 ms and all should be >0.6 ms based on neural refractoriness (Heil et al., 2007). To confirm that the recorded unit was part of the AN bundle, and not of the cochlear nucleus, several additional checks were conducted. Spike waveforms were checked for the absence of a prepotential, the response pattern to tones at 20–30 dB above threshold were checked for having a primary-like shape, and the shape of the rate-level function was required to fall into one of the three known rate-level function shapes of the AN (straight, sloping saturating, or fast saturating; Winter et al., 1990). When responses to clicks were obtained, click latency was determined and matched to our own click latency versus BF distribution of AN fibers. When any of these checks failed, the unit was excluded from further analysis.

### Vowel discrimination

Responses to vowels were included in the analysis when a minimum of 20 successful trials were recorded and a minimum of 200 spikes were evoked during vowel presentation. Responses of vowels in noise were pooled across the two different frozen background noises, but not across different speakers. For the female speaker, a total of 39 units were included in the study and in *n* = 23 of these units, responses to all vowels could be recorded and included in analyses. For the male speaker, a total of 15 units were included and in *n* = 12 units responses to all vowels were obtained. There were no units in which responses to both the female and the male speaker were recorded. Table 2 gives an overview of the number of units for each vowel and vowel combination.

**Table 2:** Numbers of units from which responses to vowels were recorded

Single vowels	/a:/	/e/	/i/
Number of units female speaker	39	33	24
Number of units male speaker	13	15	15
			
Vowel discriminations	/a:/ vs /e/	/a:/ vs /i/	/e/ vs /i/
Number of units female speaker	33	24	23
Number of units male speaker	12	12	14

**Figure 2.**
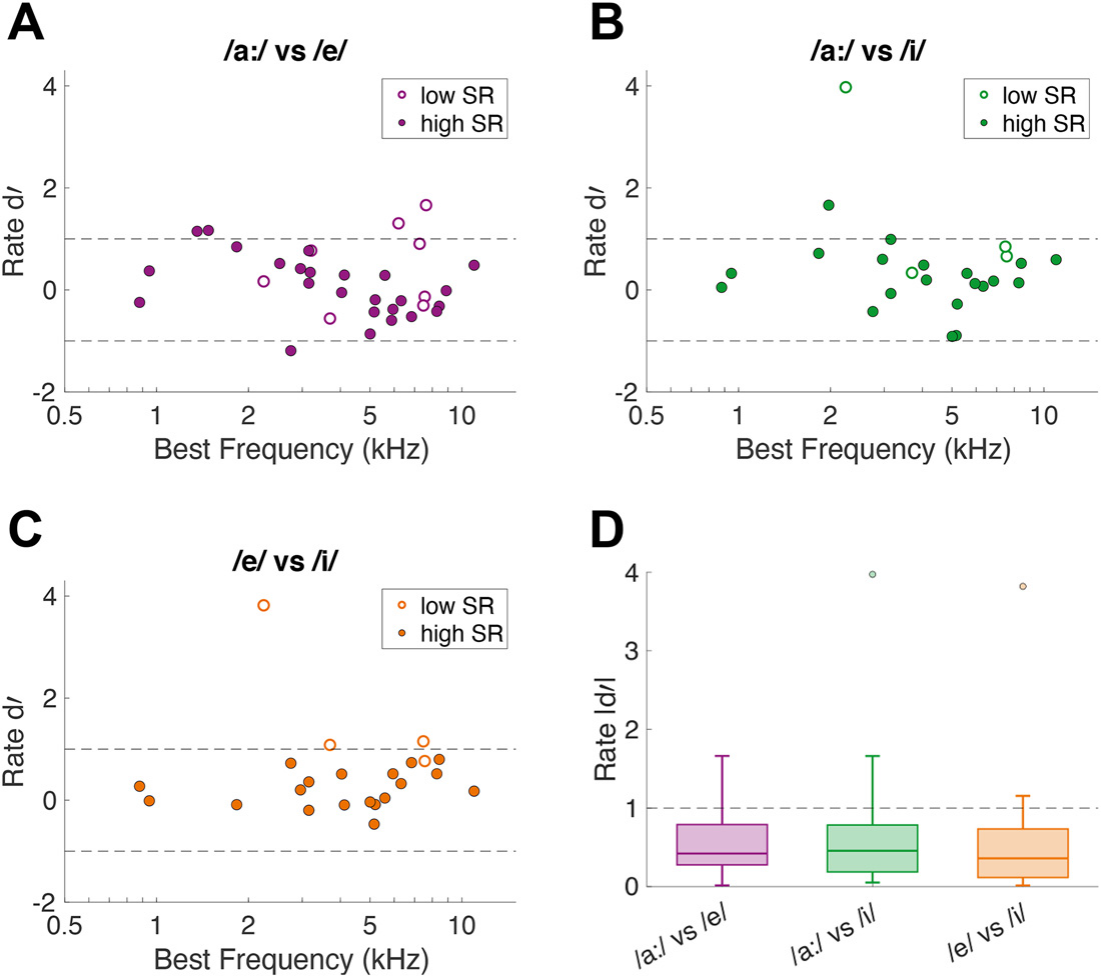
Single-unit AN-fiber vowel discrimination based on average firing rate. ***A–C***, d’ values for each unit’s firing rate in response to the two vowels that are compared, plotted as a function of the unit’s BF. Low-SR and high-SR fibers are plotted with open and closed symbols, respectively. Dashed horizontal lines indicate a neural discrimination threshold of |d’| = 1. Number of units (of those, number of low-SR units) included in the vowel comparisons are *n* = 33 (8), *n* = 24 (4), and *n* = 23 (4) for panels ***A***, ***B***, and ***C***, respectively. ***D***, Absolute d’ values of all units presented in boxplots. The dashed horizontal line indicates |d’| = 1.

**Figure 3.**
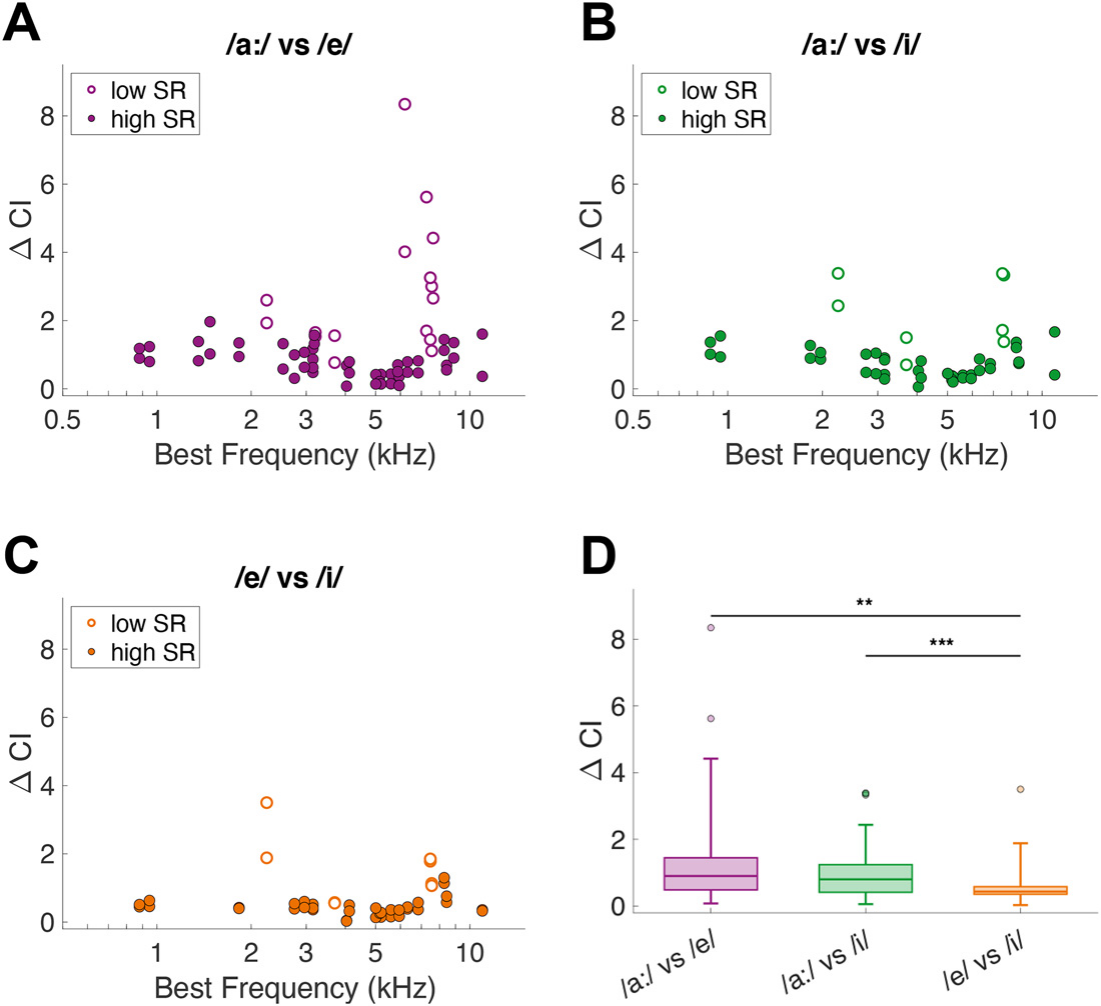
Single-unit AN-fiber vowel discrimination based on spike timing. ***A–C***, ΔCI values for each unit in response to the two vowels that are compared, plotted as a function of the unit’s BF. Low-SR and high-SR fibers are plotted with open and closed symbols, respectively. Number of units (of those, number of low-SR units) included in the vowel comparisons are *n* = 33 (8), *n* = 24 (4), and *n* = 23 (4) for panels ***A***, ***B***, ***C***, respectively. Note that there are two values per unit in this analysis. ***D***, ΔCI of all units presented in boxplots. Asterisks indicate a significant difference, based on *post hoc* paired *t* tests (**p < 0.01, ***p < 0.001).

**Figure 4.**
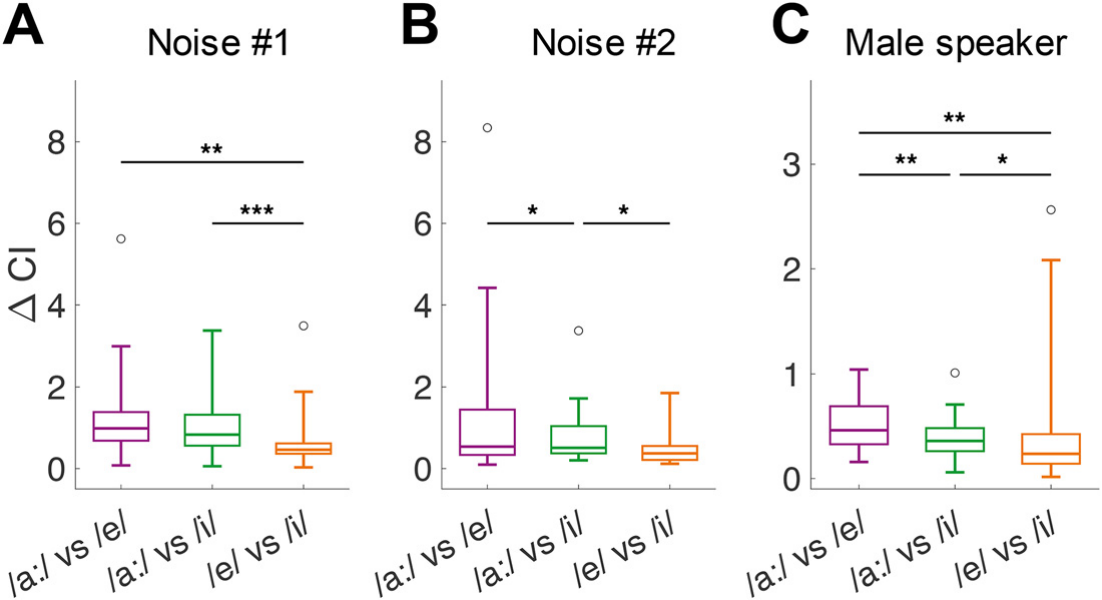
Effects of background noise and speaker-specific characteristics on spike-timing-based vowel discrimination. ***A***, ΔCI values for vowel comparisons presented against background noise #1 at 5 dB SNR, spoken by a female speaker of the OLLO database (S01F). Number of units are *n* = 20 for /a:/ vs /e/, *n* = 13 for /a:/ vs /i/, and *n* = 13 for /e/ vs /i/. ***B***, ΔCI values for vowel comparisons presented against noise #2 (5 dB SNR, S01F speaker of the OLLO database). Number of units are *n* = 13 for /a:/ vs /e/, *n* = 11 for /a:/ vs /i/, and *n* = 10 for /e/ vs /i/. ***C***, ΔCI values for vowel comparisons spoken by a male speaker of the OLLO database (S06M), presented against noise #1. Number of units are *n* = 12 for /a:/ vs /e/, *n* = 12 for /a:/ vs /i/, and *n* = 14 for /e/ vs /i/. Asterisks indicate a significant difference, based on *post hoc* paired *t* tests after the RM-ANOVA revealed a significant difference between the comparisons (*p < 0.05, **p < 0.01, ***p < 0.001).

Unit numbers are given for the responses to a single vowel (data in Figs. 6, 7, 9, 10) and for responses to a combination of two vowels (data in Figs. 2-4). Unit numbers for responses to vowels spoken by the female and male speaker are presented separately.

In total, three comparisons were made, two behaviorally easy comparisons (/a:/ vs /e/ and /a:/ vs /i/) and one behaviorally difficult comparison (/e/ vs /i/). Rate-based vowel discrimination by single-unit AN fibers was determined by calculating d-prime (d’) over the mean (µ) and SD (σ) of the firing rates in response to two vowels, according to the following equation:

$$d\prime =\frac{\mu_{1}-\mu_{2}}{\sqrt{\left(\sigma_{1}^{2}+\sigma_{2}^{2}\right)/2}}.$$

A |d’| = 1 means that the mean firing rates in response to the two vowels are 1 SD apart, which is typically considered to be a just-noticeable difference between two responses and can be used as a discrimination threshold (Green and Swets, 1974; Conley and Keilson, 1995).

Spike timing-based discrimination of vowels was based on the shuffled and crossed autocorrelograms of responses to one vowel and between two vowels, respectively (Joris, 2003; Louage et al., 2004). Autocorrelograms were constructed using a bin width of 20.48 µs and were normalized for firing rate, repetition number, bin width, and analysis window. The shortest analysis window length (for /i/, see Table 1) was applied to all vowels in this analysis. The maximum peak height of the shuffled and crossed autocorrelogram is an indication of trial-by-trial temporal similarity and is defined as the correlation index (CI) and the cross-CI (CIx), respectively. Hence, the CIx indicates the similarity of the temporal responses to the two vowels that are discriminated, with low values meaning low similarity and thus suggesting good discrimination. However, if the CI, i.e., the temporal similarity of the response to one vowel, is already low, the CIx is also expected to be low which, in this case, is meaningless re. discriminability. Therefore, the difference between CI and CIx will be taken as a temporal discrimination metric, as follows:

ΔCI=CI−CIx.

A high ΔCI value reflects temporal dissimilarity between responses to the two vowels in the comparison, whereas a low ΔCI value reflects temporally similar responses to the two vowels, regardless of the overall temporal precision of spiking of a given AN fiber.

### Dominant component scheme

An all-order interspike interval histogram (ISIH) was constructed based on spike times during presentation of the vowel (Table 1). Only intervals shorter than 31.25 ms were included in the histogram, which consisted of 256 bins, resulting in a bin width of 0.12 ms. The histogram was converted to interval rate by multiplying the probability of obtaining an interval that falls into a certain bin with the average discharge rate (Sachs and Young, 1980). A fast-Fourier transform (FFT) of the all-order ISIH revealed responses to the different frequencies. The dominant component scheme was constructed by plotting the frequency of the highest peak in the FFT of the ISIH against the unit’s BF.

### Averaged localized interval rate (ALIR)

To compute the ALIR, the average peak height of the ISIH FFT at each harmonic of f0 was calculated for all units tuned at or close to that harmonic (Sachs and Young, 1980). A range of +/− 0.5 octave around each harmonic was taken to determine for which units the ALIR was computed. This results in the following equation to compute the ALIR:

$$ALIR(k)=\frac{1}{N_{k}}\overset{N_{k}}{\underset{l=1}{\sum }}H_{kl},$$

in which *N_k_* is the number of fibers tuned within +/− 0.5 octaves of the k^th^ harmonic, *l* is the fiber number within that set of fibers, and *H_kl_* is the ISIH FFT peak height of the *l*^th^ fiber at the *k*^th^ harmonic of f0. Subsequently, the ALIR of each vowel response was plotted as a function of frequency of the f0 harmonics.

### Fluctuation amplitude profile

Fluctuation amplitude profiles were constructed using the peristimulus time histogram (PSTH) of the neural response to the vowel (Carney, 2018). To capture f0 fluctuations in the PSTH, bin width was defined as eight bins per f0 period (1/8 × f0). The amplitude of the fluctuation was determined using the coefficient of variation (CV), CV = σ_n_/µ_n_, in which *n* refers to the bin height of the PSTH in spikes per second. Subsequently, the fluctuation amplitude was also established by calculating the rate change of the PSTH, i.e., the mean absolute difference between each bin height divided by the mean bin height. CV and rate change values close to 0 indicate little to no fluctuation, i.e., putative formant frequency encoding. The fluctuation amplitude was plotted as a function of the fiber’s BF to obtain the fluctuation amplitude profile.

### Rate-based excitation pattern

The rate-based excitation pattern plots the vowel-evoked normalized rate as a function of the fiber’s BF (Sachs and Young, 1979). The average firing rate during the vowel was normalized for the fiber’s SR and saturation rate as follows:

$$Normalized\;rate=\frac{rate-SR}{saturation\;rate-SR}.$$

The saturation rate was derived from the rate-level function as the firing rate at 50 dB above threshold, according to Sachs and Young (1979).

### Statistics

Rate-based and spike-timing-based discrimination was compared between the different vowel combinations using a one-way repeated-measures ANOVA (RM-ANOVA), followed by *post hoc* paired *t* tests. The Bonferroni–Holm correction was applied to multiple comparisons. Frequency distributions of the dominant components derived from the ISIH FFTs were compared between responses to the different vowels using a Kolmogorov–Smirnov test. Statistical analyses were conducted in MATLAB using the Statistics and Machine Learning Toolbox. Data were presented separately for low-SR and high-SR fibers, with the cutoff between these populations at 18 spikes/s (Schmiedt, 1989).

## Results

### Perceptual and neural discrimination of vowels agree when using a spike timing-based but not when using a rate-based discrimination metric

Discrimination between vowels in neural responses of single AN fibers was studied using rate-based and spike timing-based discrimination metrics. To determine whether mean firing rate differed significantly between responses to different vowels, d’ based on firing rate during the vowel presentation was calculated. Most d’ values were between −1 and 1 for all three comparisons (/a:/ vs /e/, /a:/ vs /i/, and /e/ vs /i/). This indicates that for most units, the firing rate did not differ significantly between responses to the different vowels (Fig. 2*A–C*). Furthermore, low-SR and high-SR units both showed a similar (small) fraction of |d’| values > 1 for all three comparisons (Fig. 2*A–C*). The BF of units with |d’| > 1 did not correspond to formant frequencies of either vowel in the comparison. Importantly, absolute d’, averaged over all units, did not significantly differ between the perceptually easy (/a:/ vs /e/ and /a:/ vs /i/) and the difficult (/e/ vs /i/) comparisons (RM-ANOVA: *F*_(2,44)_ = 0.76, *p* = 0.47; Fig. 2*D*), indicating that the rate d’ of neural responses cannot predict perceptual vowel discrimination.

The spike timing-based neural discrimination metric was calculated using auto- and cross-correlation indices. For the resulting ΔCI, low-SR fibers achieved much higher maximal values compared with high-SR fibers, which was evident for all three comparisons (Fig. 3*A–C*). Furthermore, ΔCI values differed across BF, such that units with a BF < 3 kHz and > 8 kHz had higher ΔCI values. Most importantly, average ΔCI differed significantly between the vowel comparisons (RM-ANOVA: *F*_(2,90)_ = 16.07, *p* = 1.07 × 10^−6^; Fig. 3*D*). Specifically, the difficult comparison (/e/ vs /i/) revealed significantly lower ΔCI values compared with both perceptually easy comparisons (/a:/ vs /e/, *post hoc* paired *t* tests: *t*_(35)_ = 3.51, *p* = 0.0021; /a:/ vs /i/, *t*_(45)_ = 4.98, *p* = 2.93 × 10^−5^, Bonferroni–Holm corrected).

To examine whether this result was influenced by specific features of the background noise, we repeated the same analysis, comparing ΔCI when vowels were presented against two different background noises. Compared with the two easy comparisons, ΔCI was again lower in both conditions for the perceptually difficult comparison (/e/ vs /i/). This difference was significant for noise #1 (RM-ANOVA: *F*_(2,50)_ = 11.45, *p* = 8.03 × 10^−5^; Fig. 4*A*) and for noise #2 (RM-ANOVA: *F*_(2,38)_ = 4.82, *p* = 0.014; Fig. 4*B*). We were also interested to observe whether vowels spoken by a different speaker, with a different f0, would yield the same outcome. Therefore, we collected responses from 17 additional units to CVCs spoken by a male speaker, all presented against background noise #1. For the male speaker, too, the perceptually difficult comparison showed lower ΔCI values compared with the easy comparisons (RM-ANOVA: *F*_(2,46)_ = 11.71, *p* = 7.75 × 10^−5^; Fig. 4*C*). Additionally, the two easy comparisons were also significantly different from each other in noise #2 and for the male speaker.

**Figure 5.**
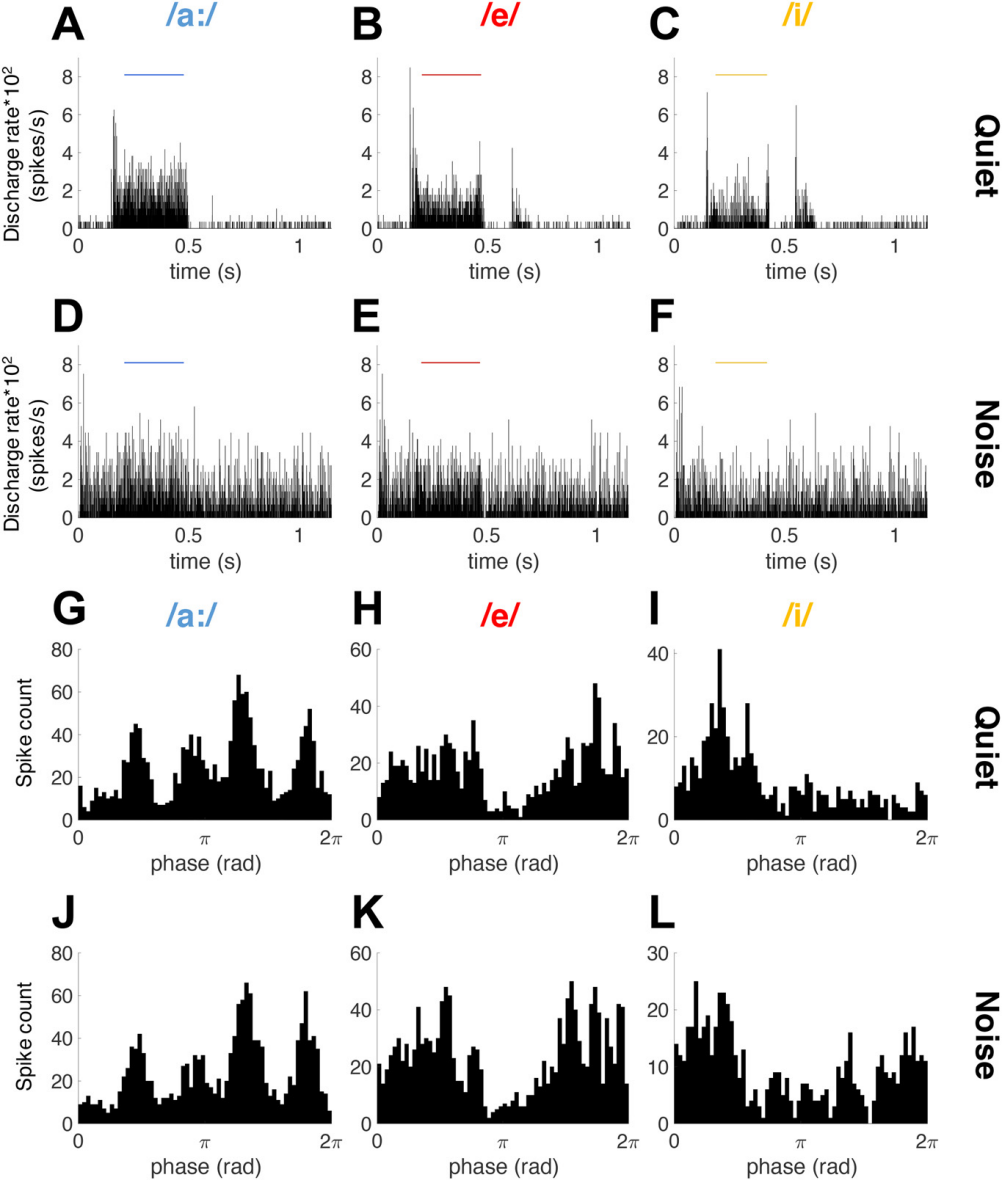
Example of one fiber responding to the three logatomes in quiet and in noise. PSTH of the response to the complete stimulus in quiet (***A–C***) and in 5 dB SNR speech-shaped ICRA1 noise (***D–F***). The vowel analysis window is indicated with a horizontal bar. Bin width is 0.49 ms for the graphs in panels ***A–F***. PH of F0 during the vowel analysis window in quiet (***G–I***) and in 5 dB SNR noise (***J–L***). PHs were plotted with 64 bins per f0 cycle. This fiber had a BF of 2.2 kHz, a threshold at 28 dB SPL, and a SR of 4.7 spikes/s. Note the remarkably little effect of noise on the shapes of the PHs after the addition of background noise (panels ***J–L***).

These results imply that spike-timing information in AN-fiber spiking patterns is important to discriminate between naturally-spoken vowels in background noise. The same is intuitively apparent by contrasting AN rate and timing responses to the naturally-spoken vowels in quiet and in the speech-shaped background noise (Fig. 5), a comparison that drastically highlights the added difficulty of discrimination in the noisy condition and the concurrent importance of spike timing. The results presented in this paragraph together suggest that formant representation of these vowels by the AN-fiber population is likely to be based on spike timing. In the following paragraphs, we will test this hypothesis.

### Spike timing-based representation schemes can differentiate formant frequencies of naturally-spoken vowels in noise

**Figure 6.**
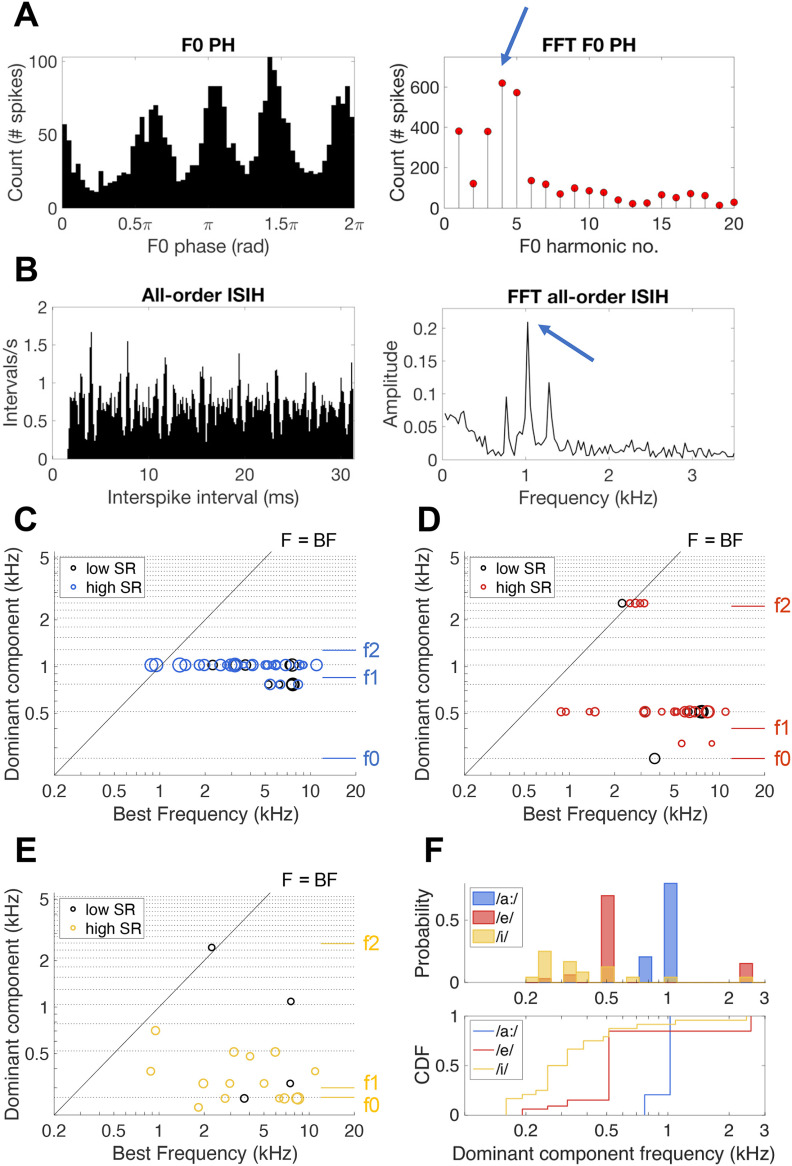
Dominant component schemes. ***A***, Response of an AN fiber to the vowel /a:/ plotted in a PH for one period of f0. The FFT of the f0 PH is plotted next to it. The arrow indicates the highest peak in the FFT of the f0 PH at the fourth harmonic of f0 and its relative peak height plotted in number of spikes. This example fiber was a high-SR fiber (46 spikes/s) with a BF at 1.3 kHz. ***B***, All-order ISIH of the same data presented in panel ***A***, accompanied by its FFT. The arrow indicates the highest peak in the FFT, at 1024 Hz, which corresponds to the frequency of the fourth harmonic of f0. ***C–E***, Dominant component schemes plotting the frequency of the highest peak in the FFT of the all-order ISIH (panel ***B***) as a function of the fiber’s BF for responses to the vowels /a:/ [panel ***C***, *n* = 39 (*n* = 10 low-SR fibers)], /e/ [panel ***D***, *n* = 33 (*n* = 8 low-SR fibers)], and /i/ [panel ***E***, *n* = 24 (*n* = 4 low-SR fibers)]. Formant frequencies are indicated on the right and the F = BF line is indicated as a solid black line. Horizontal dotted lines indicate frequencies of f0 harmonics. Symbol size reflects the height of the peak in the ISIH FFT, which is a measure of the strength of the temporal coding at the respective frequency component. ***F***, Frequency distributions of dominant components for responses to /a:/, /e/, and /i/ in blue, red, and yellow, respectively. Data are plotted in a histogram (upper panel) as well as in the form of a cumulative distribution function (CDF; lower panel).

The dominant component scheme, first proposed by Delgutte and Kiang (1984a), is an overview of the dominant frequency component in the temporal spiking response to the vowel by a population of AN fibers across different BF. The dominant component is typically derived from a Fourier transform of the f0 phase histogram (PH; Fig. 6*A*). However, with naturally-spoken vowels, f0 may change over time. Furthermore, formant frequencies can be located between two f0 harmonics, instead of precisely coinciding with an f0 harmonic. To account for this natural variability, the frequency of the largest peak in the Fourier transform of the all-order ISIH was used to construct the dominant component scheme (Fig. 6*B*).

Figure 6*C–E* shows the dominant component scheme, based on the all-order ISIH Fourier transform, of the AN-fiber responses to the three naturally-spoken vowels in noise. Formant representation in the dominant component schemes differed between the different vowels. Responses to the vowel /a:/ were all dominated by temporal responses to the third and fourth f0 harmonics, as such likely representing f1 and f2. Harmonic responses to f1 and f2 were represented by a subset of fibers for the responses to /e/ and /i/, leaving some fibers also responding to f0 or to frequencies between formants and f0 harmonics. There were no apparent differences between low-SR and high-SR units in the dominant component schemes. When comparing the frequency distribution of the dominant components, all three distributions were significantly different from each (Kolmogorov–Smirnov tests: /a:/ vs /e/, *D *=* *0.85, *p* = 6.53 × 10^−12^; /a:/ vs /i/, *D *=* *0.92, *p* = 8.07 × 10^−12^; /e/ vs /i/, *D *=* *0.64, *p* = 8.93 × 10^−6^; Fig. 6*F*). This agrees with the fact that all three vowels can be perceptually distinguished from each other (Jüchter et al., 2021). Nevertheless, out of all three comparisons, the KS statistics and p-value was smallest for the /e/ vs /i/comparison, following expectations based on perceptual discrimination measures.

**Figure 7.**
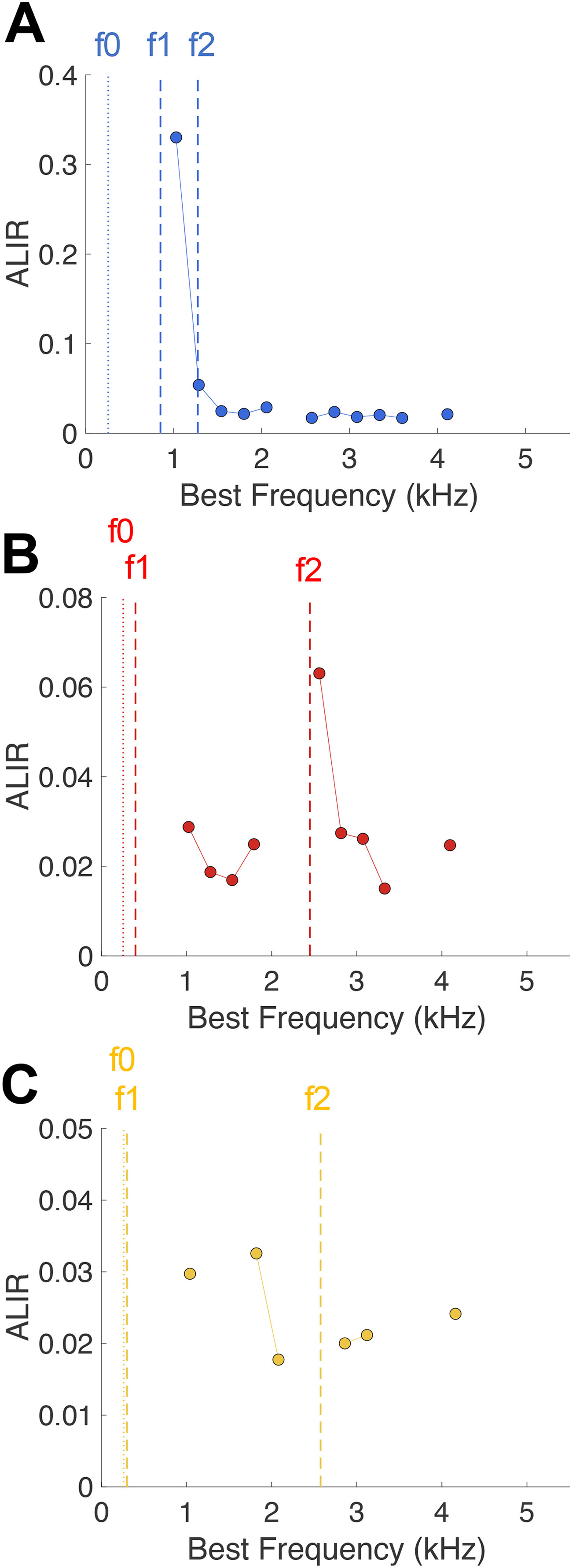
ALIR schemes. ***A–C***, ALIR schemes plotting the average ISIH FFT amplitude around each harmonic of f0 for fibers with a BF within +/− 0.5 octave of the respective f0 harmonic, for response to the vowels /a:/ (panel ***A***), /e/ (panel ***B***), and /i/ (panel ***C***). Formant frequencies are indicated by dashed lines and f0 is indicated as a dotted line for each vowel ALIR scheme.

Since one component may be dominant in many fibers, it has been proposed taking the average peak height of the f0 PH FFT or of the ISIH FFT only of fibers that are tuned at or close to the respective f0 harmonic (Sachs and Young, 1980; Voigt et al., 1982). The resulting ALSR or ALIR, respectively, is a measure derived from the peak amplitudes of the FFTs (Fig. 6*A*,*B*). At specified frequency steps around each harmonic of f0, the average of this amplitude is taken for all units tuned to this f0 harmonic +/− 0.5 octaves. Plotting the ALSR or ALIR as a function of the respective f0 harmonic reveals a spectrum that has been shown to be robust against background noise, stimulus level, and inharmonic, whispered vowels (Young and Sachs, 1979; Voigt et al., 1982; Sachs et al., 1983). Figure 7 shows the ALIRs derived from combined neural responses of low-SR and high-SR fibers to the three naturally-spoken vowels in noise. The current dataset did not cover BFs at all harmonics of f0, explaining the missing points in the ALIR. Responses to vowel /a:/ showed a dominant peak at the fourth f0 harmonic, representing formant frequencies f1 and f2 (Fig. 7*A*). The ALIR of responses to /e/ showed a trough at frequencies between f1 and f2, and a clear peak at the 10th f0 harmonic, representing f2 (Fig. 7*B*). Likely because of a lower number of data points (see Table 2), the ALIR of responses to the vowel /i/ does not represent the formant frequencies clearly. Low-SR fibers contributed only marginally to these schemes, since most low-SR fibers had a BF > 4 kHz, which is the upper frequency border of these ALIRs and also above of the phase-locking range for gerbil AN fibers (Versteegh et al., 2011).

**Figure 8.**
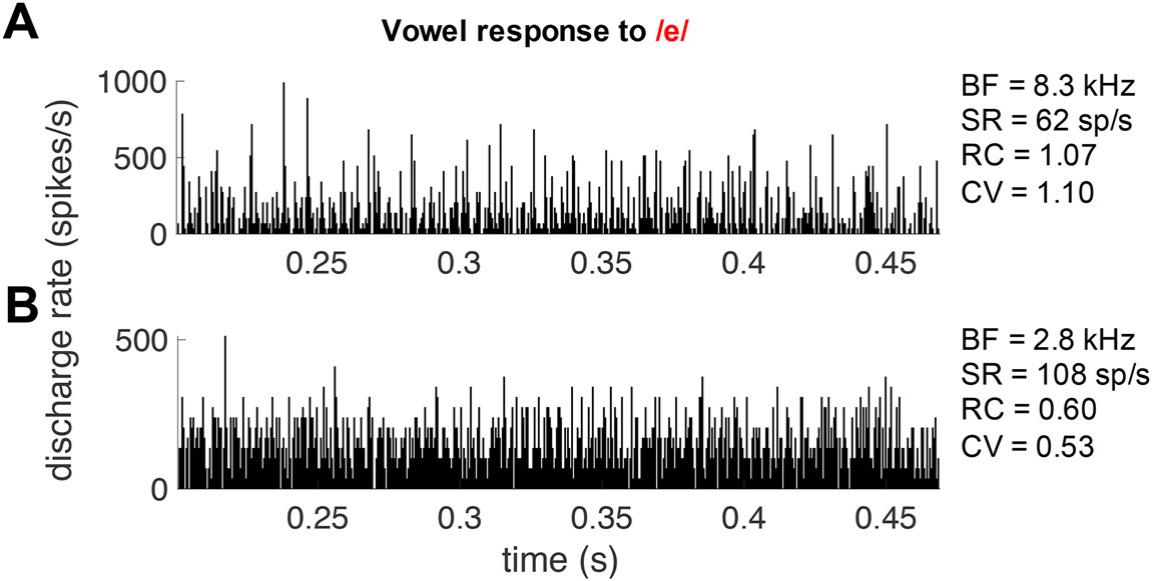
Examples of the PSTH of two fibers during the presentation of the vowel /e/. ***A***, This is an example of a highly fluctuating response. The fiber’s BF is far away from the formant frequencies of the vowel /e/ (at 8.3 kHz). ***B***, This is an example of a flat response, showing little fluctuation. The fiber’s BF is close to f2 (BF = 2.8 kHz, f2 = 2.5 kHz). Both responses derived from high-SR fibers. Fluctuation metrics CV and RC (rate change) are close to twice as high for the fluctuating (panel ***A***) compared with the flat response (panel ***B***). The *x*-axis represents the time in seconds relative to the start of the complete stimulus. Only the vowel’s analysis window is shown. Bin width is 0.49 ms (1/8 × f0). Bin height represents the average discharge rate.

**Figure 9.**
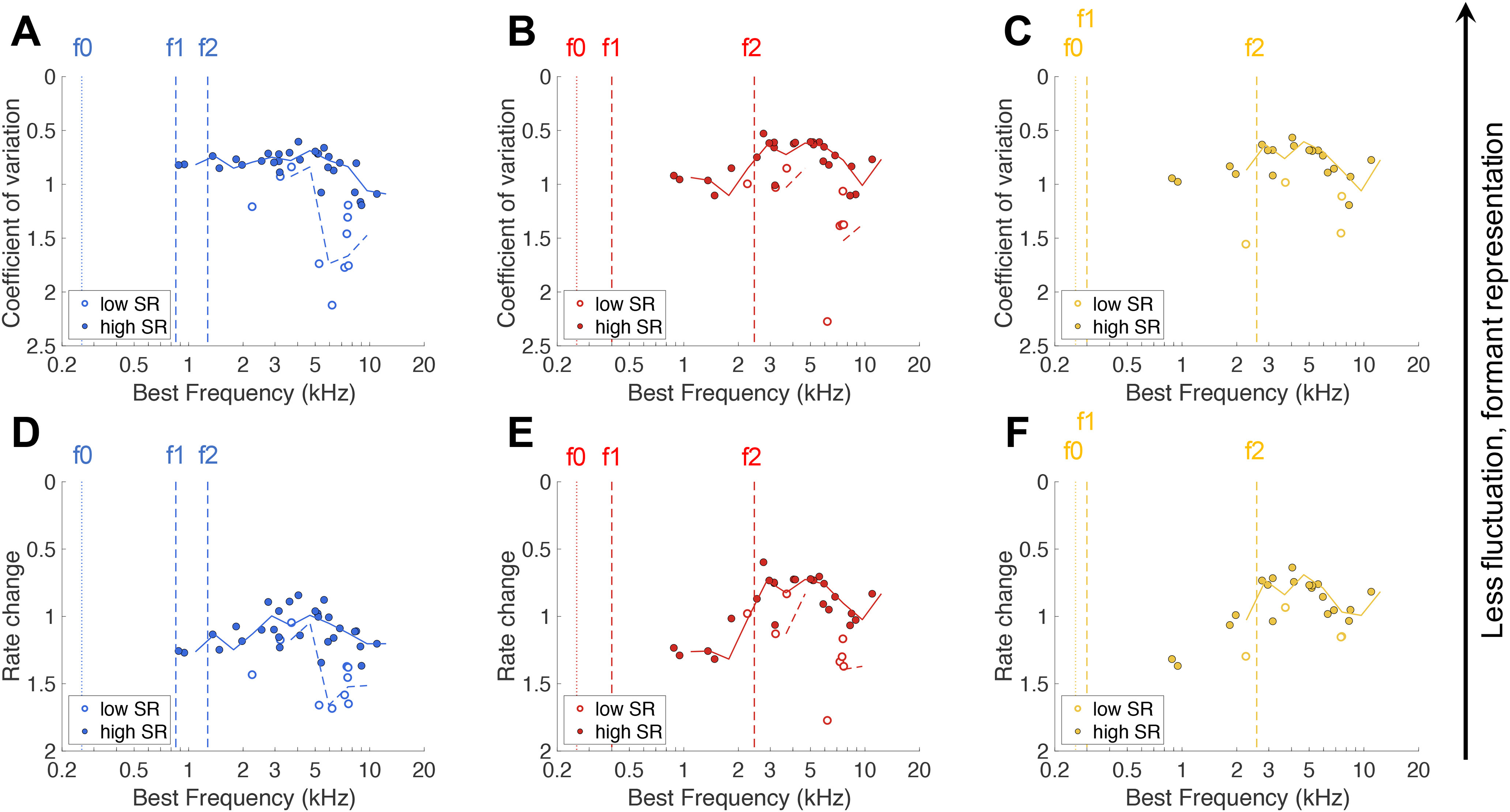
Fluctuation amplitude profiles. ***A–C***, CVs of responses to the vowels /a:/ [panel ***A***, *n* = 39 (*n* = 10 low-SR fibers)], /e/ [panel ***B***, *n* = 33 (*n* = 8 low-SR fibers)], and /i/ [panel ***C***, *n* = 24 (*n* = 4 low-SR fibers)] as a function of the fiber’s BF. ***D–F***, Rate change, calculated as the mean absolute difference between each bin height divided by the mean bin height of the PSTH, of the three presented vowels. Vowels and unit numbers for panels ***D–F*** are the same as in panels ***A–C***, respectively. Note that the ordinate is plotted in reverse direction, meaning that lower CVs and rate change values, referring to less fluctuation in the PSTH and thus putatively better formant representation, are higher in the graph. Moving averages of high-SR and low-SR fibers are plotted as solid and dashed lines, respectively. Formant frequencies are indicated by dashed vertical lines and f0 as a dotted line for each vowel.

A more recently developed representation scheme of neural vowel encoding is the fluctuation amplitude profile (Carney, 2018; Scheidiger et al., 2018). This scheme proposes that slow rate fluctuations to frequencies around f0 are strongest for fibers with a BF below, between, or above the formant frequencies, whereas fibers tuned at the formants display a more constant rate response, i.e., a less fluctuating PSTH. The degree of fluctuation was derived from the PSTH of the vowel response. Figure 8 shows an example of a fiber with high and a fiber with low fluctuation amplitudes to the vowel /e/ (Fig. 8*A*,*B*, respectively). Fluctuation amplitude was expressed as RC (rate change) and CV. Indeed, the response with visually high fluctuation amplitudes had a RC and CV value that was about twice as high compared with the response with visually low fluctuation amplitudes. The high fluctuation amplitude response was recorded from a fiber tuned far from the formant frequencies (BF = 8.3 kHz; Fig. 8*A*), whereas the low fluctuation response was recorded from a fiber tuned near f2 of the vowel /e/ (BF = 2.8 kHz; Fig. 8*B*). Both fibers had a high SR, to ensure a valid comparison, as low-SR fibers typically showed higher fluctuation amplitudes in general (see below). Fluctuation profiles were obtained by plotting the CV (Fig. 9*A–C*) and the rate change of the fiber’s vowel response (Fig. 9*D–F*) as a function of the fiber’s BF. Since less fluctuation hypothetically corresponds to better formant representation, the ordinate is plotted in reverse direction. Both methods showed similar patterns. Low-SR and high-SR fiber responses suggested two separate populations, with low-SR fibers showing higher CVs and rate change compared with the general trend of the high-SR fibers. For responses to the vowel /a:/, there was no clear formant-related peak in the fluctuation profile of the high-SR fibers, indicated by the moving average over the high-SR fibers in the CV and rate change (solid lines in Fig. 9*A*,*D*, respectively). The low-SR fibers appeared to contribute more to the fluctuation profile, by showing large fluctuations at high frequencies, but the number of low-SR fibers was too small to draw conclusions. For responses to the vowels /e/ and /i/, the moving averages of the high-SR fibers suggested a peak at f2 in the fluctuation amplitude profiles (Fig. 9*B*,*C*,*E*,*F*). No fibers with a BF at or around f1 of /e/ and /i/ were recorded. In the fluctuation profiles of /e/ and /i/, low-SR fibers similarly seemed to enhance the fluctuation profile of the high-SR fibers, but are too scarce to make conclusive statements.

### The rate-based excitation pattern does not represent formant frequencies of naturally-spoken vowels in noise

**Figure 10.**
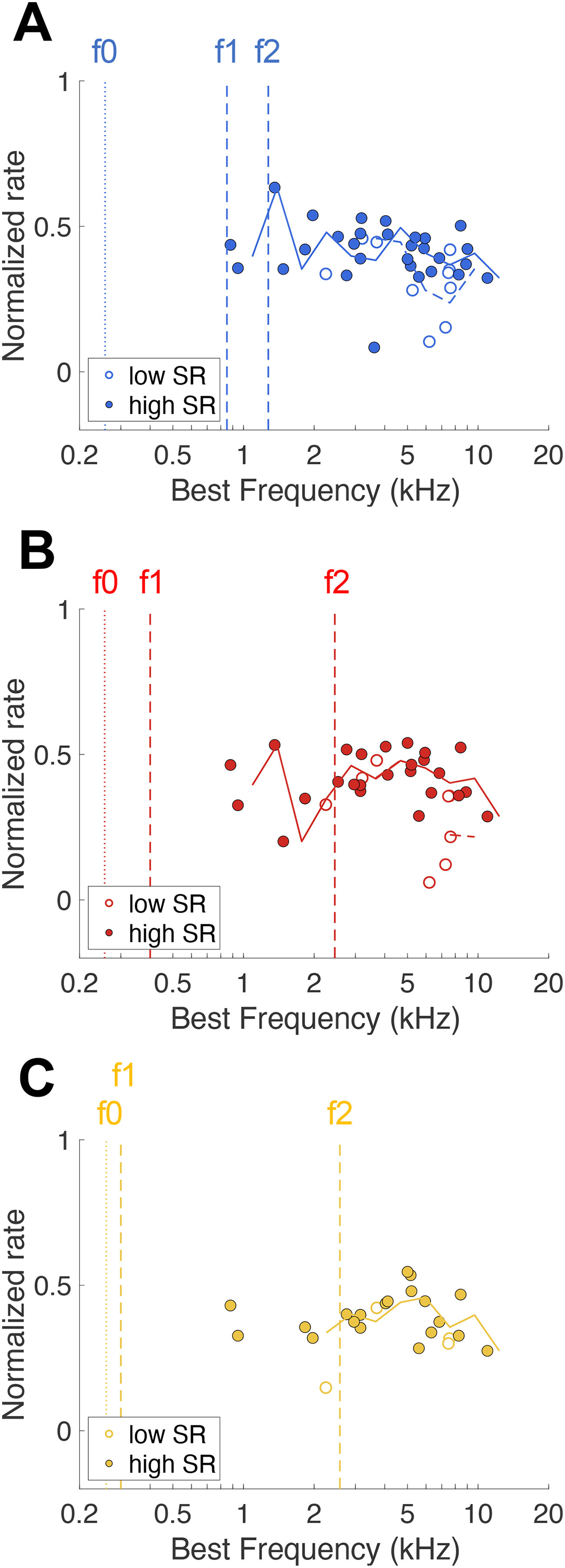
Rate-based excitation patterns. ***A–C***, Rate-based excitation patterns of AN fiber responses to the vowels /a:/ [panel ***A***, *n* = 39 (*n* = 10 low-SR fibers)], /e/ [panel ***B***, *n* = 33 (*n* = 8 low-SR fibers)], and /i/ [panel ***C***, *n* = 24 (*n* = 4 low-SR fibers)] plotted as a function of the fiber’s BF. Moving averages of high-SR and low-SR fibers are plotted as solid and dashed lines, respectively. Formant frequencies are indicated by dashed vertical lines and f0 as a dotted line for each vowel.

To cross-check for representation of the three vowels in the average firing rate response, we also plotted the normalized rate-based excitation pattern (Sachs and Young, 1979). Vowel-evoked firing rates, normalized for each fiber’s spontaneous and saturation rate, was highly variable across the fibers and did not represent formant frequencies of the naturally-spoken vowels in noise (Fig. 10*A–C*). Furthermore, there were no apparent differences in normalized rate between high-SR and low-SR fibers for any of the vowel responses.

## Discussion

The current study showed that discrimination between vowel-evoked responses of single AN fibers agreed with perceptual discrimination abilities when analyzed using a spike timing-based discrimination metric. In contrast, no differences between easy and difficult comparisons were observed when considering a rate-based discrimination metric. Consistently, spike timing-based representation schemes of the AN population response revealed representation of the vowel’s formant frequencies, whereas no such representation was apparent in a rate-based excitation pattern. Formant frequencies were differentially represented for the different vowels in the representation schemes and low-SR fibers comprised a separately responding group of fibers in their temporal responses. The findings were based on responses to vowels presented in challenging listening conditions, i.e., naturally spoken and presented in 5 dB SNR speech-shaped background noise.

### Temporal spiking information in AN fibers is suited to encode naturally-spoken vowels in background noise

Early research on the neural encoding of vowels showed that a rate-based excitation pattern, using the normalized rate as in the current study, reveals peaks at fibers tuned to formant frequencies (Sachs and Young, 1979). However, rate-based excitation patterns are not robust against a few critical situations. When vowels are presented at levels higher than 40–65 dB SPL, depending on the vowel, formant peaks disappear (Sachs and Young, 1979). Furthermore, formant peaks in the rate-based excitation patterns also disappear when vowels are presented in background noise (Sachs et al., 1983; Delgutte and Kiang, 1984b). The effects of moderate-to-high sound levels and of background noise on the rate response to vowels can be explained by two nonlinearities in the rate responses of AN fibers, namely rate saturation and two-tone suppression (Sachs and Young, 1979, 1980). Especially two-tone suppression, i.e., suppression of the firing rate to tones at BF by lower-frequency tones, was suggested to be responsible for the flattening of the rate-based excitation pattern when vowels were presented at high sound levels and in noise (Sachs et al., 1983). Our data are consistent with these findings, showing no apparent formant-related peaks in the normalized rate-based excitation pattern of vowels presented in background noise (Fig. 10). Single-fiber discrimination abilities based on rate revealed significant changes in a few fibers for all three vowel comparisons. However, these high d’ values did not appear to relate to the formant frequencies, in contrast to that shown for formant frequency shifts in synthetic vowels presented in quiet (Conley and Keilson, 1995). Importantly, average d’ values over all units did not distinguish between perceptually difficult and perceptually easy vowel discriminations (Fig. 2*D*), based on a behavioral study using the same stimuli and the same animal species (Jüchter et al., 2021). This suggests that the average firing rate of AN fibers is not crucial for encoding naturally-spoken vowels in background noise.

In contrast, discrimination of vowel responses by a spike timing-based metric did agree with expectations from behavioral studies (Fig. 3*D*). Specifically, the perceptually difficult discrimination (/e/ vs /i/) also evoked the temporally most-similar spiking patterns in AN fibers, compared with the perceptually easy discriminations (/a:/ vs /e/ and /a:/ vs /i/). In agreement with the temporally based discrimination at the single-unit level, representation schemes that were based on spike-timing patterns, i.e., the dominant component scheme, the ALIR, and the fluctuation profile, represented peaks at most formant frequencies of the naturally-spoken vowels in noise.

It has been shown previously that the dominant component scheme, as well as the ALIR, is robust against higher sound levels (Young and Sachs, 1979; Delgutte and Kiang, 1984a), as well as against background noise (Sachs et al., 1983; Delgutte and Kiang, 1984b). Both these representation schemes are based on phase-locking strength, on the frequencies that are encoded by the temporal spiking patterns, and on the BF of the fiber. Since the vowels for this study were naturally spoken, the classical representation, based on the f0 PH and its Fourier transform (Sachs and Young, 1979; Delgutte and Kiang, 1984a), was less sensitive for the higher f0 harmonics. Therefore, we used the ISIH to determine phase-locking frequencies of the single-fiber responses (Sachs and Young, 1980). This method has previously proven useful for voiceless, whispered vowels, which lack f0 and its harmonics (Voigt et al., 1982). By constructing an all-order ISIH, compared with a first-order ISIH as in most studies, the resolution of the FFT and its relevant peaks became sufficiently high. Experimental data showing fluctuation profiles of AN fiber responses to vowels have not been reported elsewhere. Models predict less fluctuation in the PSTH for fibers tuned at or close to the formant frequencies and more fluctuation for fibers tuned to frequencies in-between or above the formant frequencies (Carney, 2018; Scheidiger et al., 2018). A broad peak at f2 was observed in the fluctuation profiles of responses to vowels /e/ and /i/. Therefore, we showed for the first time that fluctuation profiles can be used to reveal some formant frequencies of spoken vowels in background noise from AN responses. Rate change appeared to be a slightly stronger metric compared with the CV, but both revealed the same patterns for all vowel responses.

All spike timing-based representation schemes applied in this study revealed formant frequencies for one or more vowels. Hence, it was unimportant precisely which temporal metric was chosen, as the principal conclusion generalizes.

### Low-SR and high-SR fibers follow separate distributions for temporally-based encoding of vowels

SRs of AN fibers vary widely between fibers, ranging from 0 to 150 spikes/s, and have been shown to correlate to physiological and morphologic properties of the fibers (Liberman, 1978, 1982; Schmiedt, 1989; Merchan-Perez and Liberman, 1996; Huet et al., 2016). In terms of rate encoding, high-SR fibers typically saturate over a narrower range of sound levels (around 20 dB) compared with low-SR fibers (Winter et al., 1990). Furthermore, low-SR fibers generally have higher thresholds than high-SR fibers (Liberman, 1978; Huet et al., 2016). These characteristics are likely to be responsible for the fact that rate-based excitation patterns of low-SR (but not high-SR) fibers are more robust, even at higher sound levels (Conley and Keilson, 1995). However, low-SR fibers do not preserve formant peaks in rate-based excitation patterns when vowels are presented in background noise (Sachs et al., 1983), which was consistent with results presented in the current study. Furthermore, low-SR fibers did not differ from high-SR fibers in terms of rate-based vowel discrimination for naturally-spoken vowels in noise.

In contrast, the temporal responses of low-SR fibers were consistently stronger than those of high-SR fibers in all vowel-evoked responses. This was apparent in the temporal discrimination values (Fig. 3) and in the fluctuation profiles (Fig. 9), with higher ΔCI and more fluctuation for low-SR fibers. This finding agrees with stronger temporal coding in low-SR fibers, as measured by the CI to broadband noise stimuli (Louage et al., 2004). Furthermore, low-SR fibers have higher maximum phase-locking values to amplitude modulations than high-SR fibers (Joris and Yin, 1992). Most low-SR fibers in this study had a BF above the maximum phase-locking frequency (i.e., >4 kHz, Versteegh et al., 2011), indicating that they were phase-locking to the lower stimulus frequencies instead of at their BF. It has been hypothesized that low-SR fibers signal mostly to the efferent feedback system, to control cochlear gain, whereas high-SR fibers encode the properties of the sound, for further processing toward perception (Carney, 2018). Our data are consistent with this theory, in which high-SR fibers follow perceptual discrimination closely and represent formant frequencies well for the spoken vowels in background noise. However, since low-SR fibers are scarce in the gerbil cochlea, especially among those with a low BF (Ohlemiller and Echteler, 1990; Huet et al., 2016), and are more difficult to hold stable for prolonged recording times than high-SR fibers, the population of low-SR fibers in the current study is perhaps too small to draw conclusions about rate and temporal vowel representation in that population.

### Formant frequencies do not all need to be accurately represented in AN fiber spiking patterns to obtain appropriate vowel discrimination

In the representation schemes that were based on temporal representations, not all formant frequencies of the spoken vowels were represented. Formants seemed to be best represented in the responses to /e/, especially in the ALIR and the dominant component scheme. In the fluctuation profile, the vowel /a:/ showed no peaks at any formant frequency, and in the ALIR of responses to /i/ formant frequencies were not represented. It is interesting that formant representation differs for the different vowels, although they were spoken by the same speaker and presented in the same background noise. In rate-based excitation patterns, formant representation of the vowel /a/ also disappears at lower sound levels compared with those of /I/ and /ε/, and f3 is less well represented for responses to /a/ (Sachs and Young, 1979). One explanation for this observation is that formant representation in the AN fiber population may depend on the amplitude of the formants relative to each other as well as relative to the background noise. As can be seen in Figure 1, the amplitude of f2 relative to the f1 amplitude in the vowel spectrum of /e/ is higher than in the vowel spectrum of /i/. Furthermore, the f2 amplitude of /e/ is well above the background noise whereas f2 of /i/ is barely above the background noise (Fig. 1*E*,*F*). On the other hand, the frequency and amplitude of f1 and f2 in the vowel spectrum of /a:/ are much closer together than in /e/ and /i/, and both are well above background noise (Fig. 1*D*) which means that they should be encoded robustly by phase-locking of many fibers to the same two harmonics. Indeed, in humans, vowel perception is not only dependent on the exact formant frequencies, but also on the relative spectral amplitude of the formants (Kiefte et al., 2010). Hence, AN fiber encoding of formant amplitude and frequency would be required for the correct identification of a specific vowel. However, the specific task investigated here involved discrimination between two vowels, which does not necessitate the correct identification of both. Indeed, based on the AN data in the current study, this is possible without a completely accurate representation of all formants.

It is an interesting question in itself how the different representation schemes suggested and explored here for the AN population might be integrated and used by the central auditory system. Note, for example, that most representation schemes convert the spike trains of each fiber into one or two dimensions, calculating one or two outcome measures from the (temporal) information in the spike train. It is convenient to reduce dimensionality when comparing between responses from fiber populations, vowel responses, or between different conditions, such as with hearing loss. However, each AN fiber branches and projects to many different cell types in the cochlear nucleus. Each cell type extracts different properties from the same spiking pattern, depending on the amount of convergence from different AN fibers, as well as on the physiological and morphologic properties of the cell (Oertel, 1991). It is therefore possible that additional temporal information in the spike trains was available but was not extracted by the applied representation schemes.

In conclusion, this study showed that naturally-spoken vowels in noise were discriminable based on temporal features of AN fiber spike trains but not on the basis of mean discharge rates. This was true both at the single-unit level, contrasting spike trains of the same fiber in response to two different vowels, and at the level of the AN population, looking at representation schemes such as the ALIR, dominant component scheme, and fluctuation profiles. Using perceptual discrimination data of the same species to the same stimuli enabled us to disentangle important aspects of neural vowel encoding in the AN.

## Synthesis

Reviewing Editor: Christine Portfors, Washington State University Decisions are customarily a result of the Reviewing Editor and the peer reviewers coming together and discussing their recommendations until a consensus is reached. When revisions are invited, a fact-based synthesis statement explaining their decision and outlining what is needed to prepare a revision will be listed below. The following reviewer(s) agreed to reveal their identity: Victor Adenis.

The reviewers agree that this manuscript adds to the literature by highlighting important aspects of peripheral speech encoding. It is especially interesting that the authors used natural vowels instead of the classical synthesized ones, in a challenging environment. The importance of spike-timing pattern over rate to discriminate sounds in challenging environment is well exposed and builds a strong case to support the theory that discrimination relies (or partly relies) on the vowel’s formant frequencies.

The potential observation of two different contributions of the High- and Low-SR peripheral neural population to vowel discrimination is also important. The manuscript can be improved by adding a stronger rationale and providing additional details in the results.

Main comments:

First, the main argument to select the 3 vowels is related to behavioral performances that are not disclosed in this article. Having a quick description of the behavioral task leading to this choice of CVC and/or a summary of the results would help as it seems a little bit arbitrary in this MS.

Second, in several figures, three Ns are usually reported, one for each vowel presented or one for each comparison. It is unclear how many units in total are part of the study and how each sample is built. For example in Fig2 (d’), the authors give N(a vs. e)= 33, N(a vs. i)=24 and N(e vs. i)=23. How many of these fibers are common to each N? Did you observed fibers that responded to the three vowels (so that it is technically in each N) or only one (and thus excluded from this particular comparison but still part of a later sample)? A census table might help or at least the mention of the total number of fiber incorporated in the study.

In the same vein, as the High- and Low-SR populations seems to contribute differently to the discrimination, it would be good to report the n separately, especially in the legends of figures that make the distinction between each population. Also, why is this distinction absent of fig 6 (Dominant Component)? Would it not be interesting to see where Low and High SR fibers are? It might also strengthen the authors’ argument on L. 351 to 353 that “In general, dominant component schemes looked most similar between vowel responses to /e/ and /i/ ... following expectations based on perceptual discrimination measures”. I would agree that the three schemes are different but I presently fail to see how /e/ and /i/ are necessarily identical enough to be in agreement with the behavioral results. It would be good to provide a more statistical argument to support this claim given that DC is one of the main arguments of the MS.

Finally a minor comment, one of the last point discussed in the present MS on line 517-519 is that formant representation in the AN fibers would rely on the relative amplitude of the formants and the figure 1 is given as an example where the f2 of /e/ has a higher relative amplitude than f2 of /a/ and /i/. It is difficult to see solely on the basis of fig 1 as the Y-Axis is without value and the difference subtle because of the size of the figure.

## Author Response

Response to the editor and reviewers

The reviewers agree that this manuscript adds to the literature by highlighting important aspects of peripheral speech encoding. It is especially interesting that the authors used natural vowels instead of the classical synthesized ones, in a challenging environment. The importance of spike-timing pattern over rate to discriminate sounds in challenging environment is well exposed and builds a strong case to support the theory that discrimination relies (or partly relies) on the vowel’s formant frequencies.

The potential observation of two different contributions of the High- and Low-SR peripheral neural population to vowel discrimination is also important. The manuscript can be improved by adding a stronger rationale and providing additional details in the results.

Main comments:

First, the main argument to select the 3 vowels is related to behavioral performances that are not disclosed in this article. Having a quick description of the behavioral task leading to this choice of CVC and/or a summary of the results would help as it seems a little bit arbitrary in this MS.

A: A short description of the behavioral task was included at the end of the Introduction in the revised manuscript on lines 113 - 119.

Second, in several figures, three Ns are usually reported, one for each vowel presented or one for each comparison. It is unclear how many units in total are part of the study and how each sample is built. For example in Fig2 (d’), the authors give N(a vs. e)= 33, N(a vs. i)=24 and N(e vs. i)=23. How many of these fibers are common to each N? Did you observed fibers that responded to the three vowels (so that it is technically in each N) or only one (and thus excluded from this particular comparison but still part of a later sample)? A census table might help or at least the mention of the total number of fiber incorporated in the study.

A: A table has been inserted in the Methods section indicating the number of units for each vowel and vowel combination. Furthermore, a description of total number of units has been added to the revised manuscript (lines 224 - 234).

In the same vein, as the High- and Low-SR populations seems to contribute differently to the discrimination, it would be good to report the n separately, especially in the legends of figures that make the distinction between each population. Also, why is this distinction absent of fig 6 (Dominant Component)? Would it not be interesting to see where Low and High SR fibers are? It might also strengthen the authors’ argument on L. 351 to 353 that “In general, dominant component schemes looked most similar between vowel responses to /e/ and /i/ ... following expectations based on perceptual discrimination measures”. I would agree that the three schemes are different but I presently fail to see how /e/ and /i/ are necessarily identical enough to be in agreement with the behavioral results. It would be good to provide a more statistical argument to support this claim given that DC is one of the main arguments of the MS.

A: For all figures where low- and high-SR fibers are presented separately, the total number of units and number of low-SR units have been inserted in the figure legends.

Figure 6 has been adjusted so that low- and high-SR units are presented separately in the dominant component schemes.

A more statistical argument, including an extra panel in Figure 6, has been added to compare the three dominant component schemes (see lines 372 - 379).

Finally a minor comment, one of the last point discussed in the present MS on line 517-519 is that formant representation in the AN fibers would rely on the relative amplitude of the formants and the figure 1 is given as an example where the f2 of /e/ has a higher relative amplitude than f2 of /a/ and /i/. It is difficult to see solely on the basis of fig 1 as the Y-Axis is without value and the difference subtle because of the size of the figure.

A: We agree that the argument has not been well made in the discussion. We meant to say that the amplitude of f2 relative to f1 is larger in /e/ compared to /i/. Furthermore, the absolute amplitude of f2 above the noise is also larger in /e/ compared to /i/. This has now been added to the discussion (see lines 545 - 553 of the revised manuscript).
